# *KAT6B*-related disorder in a patient with a novel frameshift variant (c.3925dup)

**DOI:** 10.1038/s41439-019-0085-3

**Published:** 2019-12-13

**Authors:** Yo Hamaguchi, Mikihiro Aoki, Satoshi Watanabe, Hiroyuki Mishima, Koh-ichiro Yoshiura, Hiroyuki Moriuchi, Sumito Dateki

**Affiliations:** 1 Department of Pediatrics, National Hospital Organization Nagasaki Medical Center, Omura, Japan; 2 Department of Human Genetics, Nagasaki University Graduate School of Biomedical Sciences, Nagasaki, Japan; 3 Departments of Pediatrics, Nagasaki University Graduate School of Biomedical Sciences, Nagasaki, Japan

**Keywords:** Neurodevelopmental disorders, Next-generation sequencing

## Abstract

Heterozygous pathogenic variants in the *KAT6B* gene, which encodes lysine acetyltransferase 6B, have been identified in patients with congenital rare disorders, including genitopatellar syndrome and Say-Barber-Biesecker-Young-Simpson syndrome. Herein, we report another Japanese patient with a *KAT6B*-related disorder and a novel de novo heterozygous variant in exon 18 of *KAT6B* [c.3925dup, p.(Glu1309fs*33)], providing further evidence that truncating variants in exon 17 and in the proximal region of exon 18 are associated with genitopatellar syndrome-like phenotypes.

Genitopatellar syndrome (GPS) is a rare autosomal dominant disorder characterized by patellar hypoplasia/agenesis, urogenital anomalies, congenital flexion contractures of the large joints, microcephaly, agenesis of corpus callosum, and hydronephrosis (OMIN #606170). Heterozygous de novo pathogenic variants in the lysine acetyltransferase 6B (*KAT6B*) gene have been reported as a cause of GPS^1^. Heterozygous truncating pathogenic variants of *KAT6B* are also associated with Say-Barber-Biesecker-Young-Simpson syndrome (SBBYSS) (OMIM#603736), which is characterized by long thumbs, great toes, an immobile mask-like face, blepharophimosis/ptosis, and lacrimal duct anomalies. Although GPS and SBBYSS were originally considered independent clinical entities, they are often indistinguishable and share some common phenotypes, such as delayed development, intellectual disability, congenital heart defects, thyroid dysfunction, and/or genital anomalies. Therefore, they are collectively called *KAT6B*-related disorders^1,2^. Herein, we report an additional Japanese patient with a *KAT6B*-related disorder and a novel heterozygous frameshift variant of *KAT6B*.

This male Japanese patient was born at 37 weeks of gestation as the first child of non-consanguineous phenotypically normal parents. During pregnancy, fetal echography showed fetal growth restriction, enlargement of the bilateral cerebral ventricles, and bilateral hydronephrosis. At birth, his length was 44.5 cm (−1.2 standard deviation [SD]), his weight was 2.12 kg (−1.6 SD), and his head circumference was 28.5 cm (−2.8 SD). He had distinct facial features, with a depressed nasal bridge, a bulbous nose, micrognathia, and low-set ears. He also had flexion contractures of the hips and knees, overlapping of the left toes, exostoses on the right foot, scrotal hypoplasia, and cryptorchidism (Fig. 1a). Cardiac echography showed patent ductus arteriosus. Brain magnetic resonance imaging (MRI) revealed agenesis of the corpus callosum (Fig. 1b). A skeletal survey showed bilateral radioulnar synostosis (Fig. 1c). At 4 months of age, the patient was diagnosed with bilateral patellae were revealed by knee MRI. Primary hypothyroidism, which was not detected by neonatal mass screening, was identified at 55 days of age (serum thyroid-stimulating hormone, 37.2 μIU/ml [normal range: 0.6–5.9]; free thyroxine, 0.88 ng/dl [1.37–1.51]; free triiodothyronine, 3.09 pg/ml [4.3–5.1]. Thyroid ultrasonography showed tolerably located thyroid glands of normal size. Levothyroxine sodium treatment promptly normalized thyroid function. The patient’s karyotype was normal, and urine steroid profile analysis did not show a pattern characteristic of cytochrome P450 oxidoreductase deficiency. At the final examination at 10 months of age, his height was 67.3 cm (−2.2 SD), and his weight was 7030 g (−2.0 SD). He had hypotonia, and his motor and mental development was severely delayed (DQ 55).

**Fig. 1:**
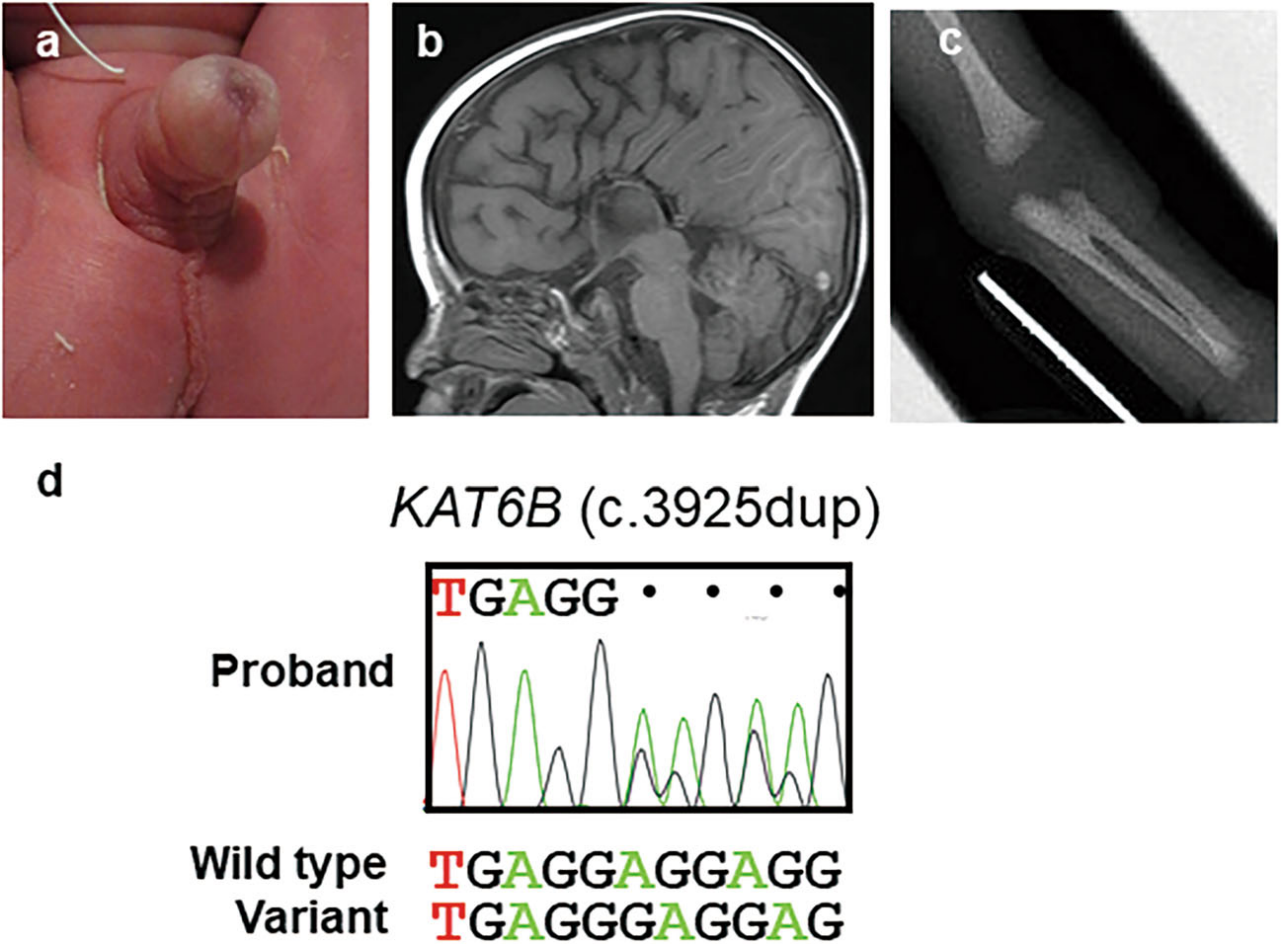
Clinical and genetic presentations of our case. **a** Cryptorchidism, **b** agenesis of the corpus callosum, and **c** radioulnar synostosis. **d** An electropherogram of the *KAT6B* gene in the proband generated by direct sequencing shows a heterozygous frameshift variant in exon 18 [c.3925dup, p.(Glu1309fs*33)].

This study was approved by the Institutional Review Board of Nagasaki University Graduate School of Biomedical Sciences. Since clinical assessment alone did not lead to a conclusive diagnosis, we sought to identify disease-causing pathogenic variants with a trio whole-exome sequencing (WES) strategy using a SureSelect Human All Exon V5 (Agilent Technologies, Santa Clara, CA, USA) on a HiSeq 2500 platform (Illumina, San Diego, CA, USA). Written informed consent was obtained from the parents. DNA was obtained from peripheral blood samples from the patient and his parents. The reads in FASTQ format were aligned to the human reference genome using NovoAlign version 3.0 (http://www.novocraft.com/). Trio-based genomic variation information was detected by the Genome Analysis Toolkit version 3.4–46^3^. Subsequently, *de novo*, homozygous, and X-linked variations were extracted and annotated by ANNOVAR software^4^; in this process, variants with an allele frequency >0.5% in the Exome Aggregation Consortium (http://exac.broadinstitute.org/), NHLBI GO Exome Sequencing Project (http://evs.gs.washington.edu/EVS/), Human Genetic Variation Database^5^ (http://www.hgvd.genome.med.kyoto-u.ac.jp), or 3.5 KJPN database of Tohoku Medical Megabank^6^ (https://jmorp.megabank.tohoku.ac.jp/201902/) were excluded. Heterozygous variations with the same annotation in GENCODE v19 were also extracted to detect compound heterozygous variants. Each variant was confirmed via Sanger sequencing using a BigDye terminator and 3130xl genetic analyzer (Applied Biosystems, Carlsbad, CA, USA).

Through these investigations, we identified several candidate variants. Of these, a de novo heterozygous variant in *KAT6B* (c.3925dup, NM_012330.3) was proposed as the best candidate based on WES data and the Online Mendelian Inheritance in Man database of known diseases (www.omim.org). The 1-bp duplication in exon 18 of *KAT6B* was predicted to cause a frameshift at codon 1309 of *KAT6B*, resulting in termination at codon 1342 of the last exon (exon 18) [p.(Glu1309Glyfs*33), NP_036462]; this prediction indicates that the frameshift pathogenic variant can escape nonsense-mediated mRNA decay (NMD) and likely produces a truncated protein lacking the distal section of the acidic domain and the entire C-terminal transcription activation domain (Fig. 2)^7^.

**Fig. 2:**
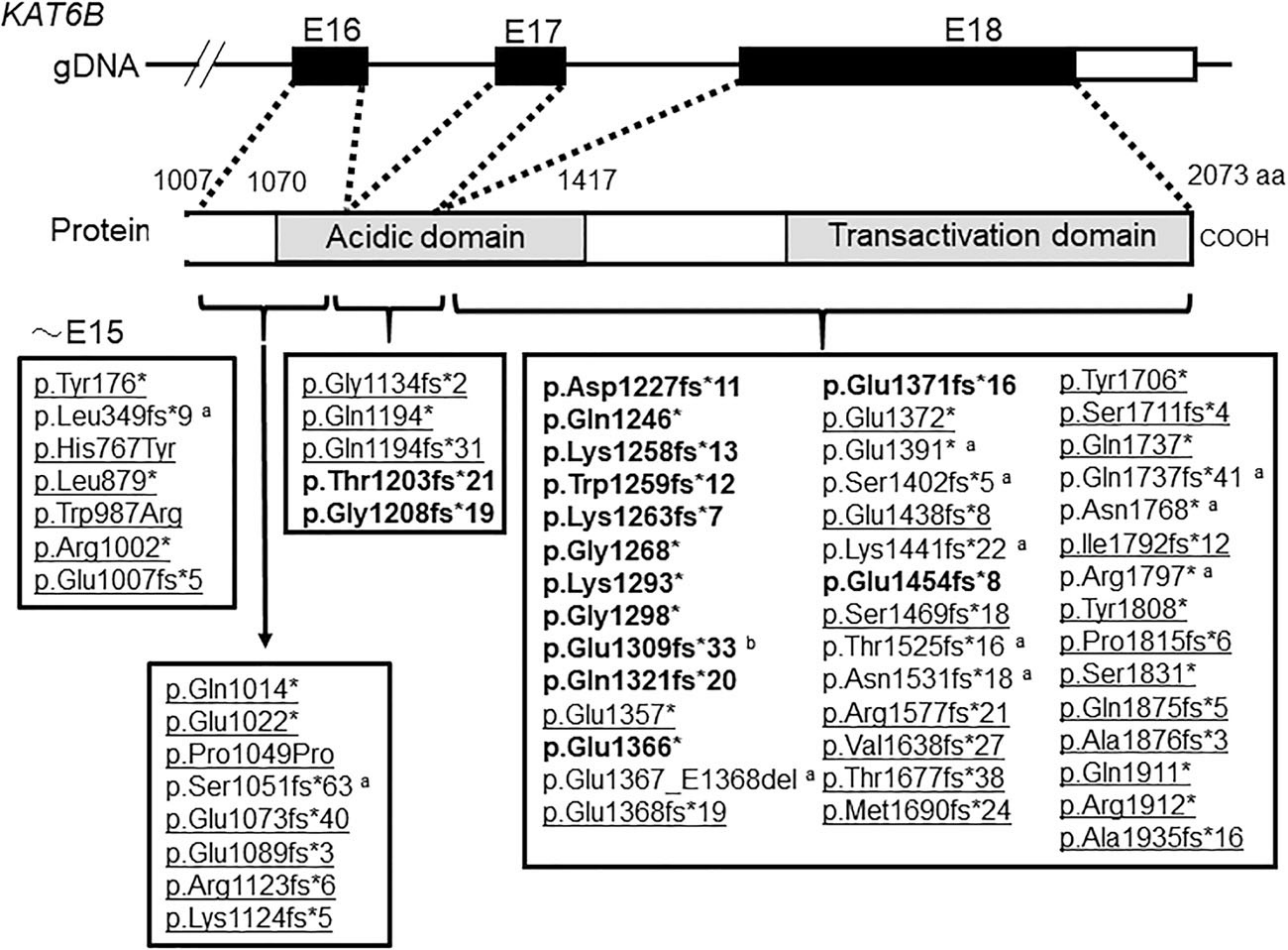
The structure of the C-terminal region of *KAT6B* and the position of the pathogenic variants associated with *KAT6B*-related disorders. The black and white boxes on genomic DNA (gDNA) denote the coding regions of exons 16–18 and the untranslated region, respectively. The variants leading to GPS and SBBYSS phenotypes are shown in bold and underline, respectively. **a** Variants with mixed or overlapping phenotypes. **b** Variant in the present patient.

To date, at least 62 pathogenic variants have been reported in patients with *KAT6B*-related disorders, which have a broad clinical spectrum, including the phenotypes of GPS and SBBYSS^1,2,8^. In this regard, several findings are noteworthy regarding genotype–phenotype correlations. GPS-specific phenotypes, such as patellar hypoplasia/agenesis, congenital flexion contractures of the large joints, microcephaly, hydronephrosis, and agenesis of the corpus callosum, have been identified in patients with truncating pathogenic variants between the distal region of exon 17 and the proximal region of exon 18 (Fig. 2). On the other hand, most of the truncating variants in the distal region of exon 18 have been identified in patients with SBBYSS-specific phenotypes, such as long thumbs, great toes, blepharophimosis/ptosis, and lower-extremity joint stiffness. In addition, more proximal truncating variants, which are predicted to undergo NMD, have been associated with milder phenotypes due to haploinsufficiency. These data and data on the present case indicate that truncated KAT6B proteins that lack the C-terminal transactivation domain but retain the proximal region of the acidic domain escape NMD and are strongly associated with GPS phenotypes.

The clinical features of the present patient highlight two interesting findings. First, the patient exhibited bilateral radioulnar synostosis, which has been reported in only one previous patient with GPS, and this previous patient had an unknown *KAT6B* genotype^9^. The present case is the first report of a genetically confirmed *KAT6B*-related disorder with bilateral radioulnar synostosis. Second, our patient presented with primary hypothyroidism, which was fortuitously diagnosed at 55 days of age. Thyroid abnormalities are often observed in patients with *KAT6B*-related disorders^10^. However, the severity and onset of the phenotype seem to be variable^11–15^. Indeed, the present patient had normal TSH levels at the time of neonatal mass screening. Hypothyroidism was diagnosed at 14 years of age in another patient with a *KAT6B*-related disorder^16^. Although the pathological mechanisms underlying the thyroid abnormalities remain to be determined, thyroid function should be frequently examined in patients with *KAT6B*-related disorders.

In conclusion, our study provides further evidence that heterozygous truncating pathogenic variants in the distal region of exon 17 and proximal region of exon 18 in *KAT6B* cause GPS phenotypes. Further studies are needed to determine the clinical spectrum of *KAT6B*-related disorders and the pathogenesis of *KAT6B* variants.

## Data Availability

The relevant data from this Data Report are hosted at the Human Genome Variation Database at 10.6084/m9.figshare.hgv.2795.
